# Lipidomics Reveals Reduced Inflammatory Lipid Species and Storage Lipids after Switching from EFV/FTC/TDF to RPV/FTC/TDF: A Randomized Open-Label Trial

**DOI:** 10.3390/jcm9051246

**Published:** 2020-04-25

**Authors:** Adrian Curran, Anna Rull, Jordi Navarro, Judit Vidal-González, Mario Martin-Castillo, Joaquin Burgos, Vicenç Falcó, Esteban Ribera, Ariadna Torrella, Bibiana Planas, Joaquim Peraire, Manuel Crespo

**Affiliations:** 1 Infectious Diseases Department, Hospital Universitari Vall d’Hebron, 08035 Barcelona, Spain; jnavarro@vhebron.net (J.N.); jburgos@vhebron.net (J.B.); vfalco@vhebron.net (V.F.); eribera@vhebron.net (E.R.); 2 Vall d’Hebron Institut de Recerca (VHIR), 085035 Barcelona, Spain; jvidal@vhebron.net (J.V.-G.); mariomcdmvl@gmail.com (M.M.-C.); atorrela@vhebron.net (A.T.); bplanasribas@gmail.com (B.P.); 3 Hospital Universitari de Tarragona Joan XXIII, IISPV, Universitat Rovira i Virgili, 43005 Tarragona, Spain; anna.rull@iispv.cat (A.R.); jperaire@comt.es (J.P.); 4 Department of Internal Medicine - Hepatology, Hospital Universitari Vall d’Hebron (HUVH), 08035 Barcelona, Spain; 5 Internal Medicine Department. Complexo Hospitalario Universitario de Vigo; IIS Galicia Sur, 36312 Vigo, Spain; manuelcrespocasal@gmail.com

**Keywords:** lipidomics, NAFLD, efavirenz, rilpivirine, switch

## Abstract

HIV and antiretroviral therapy affect lipid metabolism. Lipidomics quantifies several individual species that are overlooked using conventional biochemical analyses, outperforming traditional risk equations. We aimed to compare the plasma lipidomic profile of HIV patients taking efavirenz (EFV) or rilpivirine (RPV). Patients ≥ 18 years old on EFV co-formulated with emtricitabine and tenofovir disoproxil fumarate (FTC/TDF) with HIV-RNA < 50 copies/mL for ≥6 months were randomized to continue EFV/FTC/TDF (n = 14) or switch to RPV/FTC/TDF (n =15). Lipidomic analyses conducted by mass spectrometry (MS) were performed at baseline and after 12 and 24 weeks. OWLiver^®^ Care and OWLiver^®^ tests were performed to estimate the presence of fatty liver disease (NAFLD). No significant differences (83% male, median age 44 years, 6 years receiving EFV/FTC/TDF, CD4^+^ count 740 cells/mm^3^, TC 207 [57 HDL-C/133 LDL-C] mg/dL, TG 117 mg/dL) were observed between the groups at baseline. Significant reductions in plasma lipids and lipoproteins but increased circulating bilirubin concentrations were observed in patients who switched to RPV/FTC/TDF. Patients on RPV/FTC/TDF showed a decrease in the global amount of storage lipids (-0.137 log_2_ [fold-change] EFV vs. 0.059 log_2_ [fold-change] RPV) but an increase in lysophosphatidylcholines (LPCs) and total steroids. Compared with EFV, RPV increased metabolites with anti-inflammatory properties and reduced the repository of specific lipotoxic lipids.

## 1. Introduction

The new era of antiretroviral therapy (ART) led to a progressive increase in life expectancy and decreased rates of opportunistic infections in people living with HIV (PLWH) [1,2], but other comorbidities not historically related to HIV have become more frequent, especially cardiovascular disease (CVD). The underlying pathogenesis of CVDs in PLWH is complex and incompletely understood, where both the HIV infection itself and ART interact with traditional, genetic, and environmental risk factors [3,4,5]. In fact, HIV infection and ART have direct effects on adipose tissue and liver function with subsequent impacts on the lipid profile [5]. Thus, it is not surprising that non-alcoholic fatty liver disease (NAFLD) is often observed in PLWH and is linked to CVD complications [6]. 

Guidelines favour the early initiation of ART in PLWH to ensure sustained HIV suppression and improved immune function. This should be balanced against the risks of lifelong drug intake with potential long-term side effects; thus, it is important that ART has the best tolerability profile. In this regard, switching from a given ART regimen to a more lipid-friendly regimen represents an option to improve lipid abnormalities and to reduce metabolic complications in PLWH in daily clinical practice. Usually, ART consists of two nucleos(t)ide analogue reverse transcriptase inhibitors (NRTIs) plus a third agent, such as an integrase strand transfer inhibitor (INSTI), a protease inhibitor (PI), or a nonnucleoside reverse transcriptase inhibitor (NNRTI). Currently, INSTIs are the most commonly used third drug in treatment-naïve patients, but in many patients, PIs and NNRTIs are still used. Furthermore, INSTIs have recently been associated with weight gain in some patients [7], the clinical impact of which has yet to be determined. Among the NNRTIs, efavirenz (EFV) had been the gold standard for years and the efavirenz (EFV)/emtricitabine (FTC)/tenofovir disoproxil fumarate (TDF) combination is still considered a first-line treatment option in some guidelines in certain clinical situations due to its efficacy, tolerability, and availability [8,9]. However, EFV could cause toxicity in neurons and hepatocytes and mitochondrial dysfunction, which can lead to adverse neurological and psychiatric events and increased serum lipid levels [10,11,12]. For that reason, clinical trials have evaluated the safety, tolerability, and laboratory abnormality impacts of switching from EFV to rilpivirine (RPV) co-formulated with TDF/FTC or tenofovir alafenamide (TAF)/FTC [13,14,15,16,17]. RPV demonstrated noninferiority with fewer side effects than EFV [13,17], including lower increases in lipid levels and fewer cases of dyslipidaemia requiring treatment [14,15,16]. Although this is well established, the underlying mechanistic pathways are not fully known, hence the need for further research. Thus, a better understanding of the biological mechanism underlying lipid abnormalities in PLWH could enable us to develop personalized therapies that will balance the efficacy and tolerability of ART with a reduced incidence of CVD and other associated metabolic complications, such as NAFLD. 

Plasma lipidomic profiling, defined as the measurement of hundreds of lipid species by mass spectrometry (MS), offers a more sensitive and powerful analysis to detect underlying changes in lipid homeostasis that are undetectable using conventional laboratory measurements. In fact, recent studies in ART-treated [18,19,20] and ART-naïve HIV positive participants [21] offered additional insights into the effect of both HIV infection and ART on lipid classes and species that could be related to CVD. However, lipidomic profiling data in PLWH are still scarce and studies comparing the effects of different ART therapies are required to develop more accurate predictive models for metabolic events. Thus, this study aims to evaluate the effects of switching from EFV to RPV co-formulated with TDF/FTC compared to maintaining treatment with EFV/FTC/TDF on the plasma lipidome.

## 2. Patients and Methods 

### 2.1. Study Design and Participants

**Figure 1:**
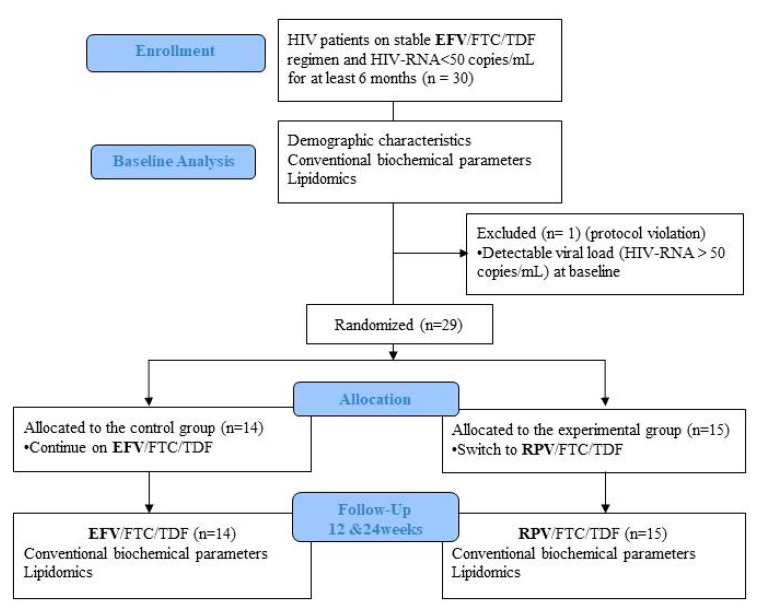
Flow diagram of participants throughout the course of the study. Abbreviations: EFV, Efavirenz; FTC, Emtricitabine; TDF, Tenofovir Disoproxil Fumarate; RPV, Rilpivirine.

This is a pilot randomized, open-label, phase IV clinical trial (EudraCT number: 2015–002319-13) conducted at the Infectious Diseases Department of the Hospital Universitari Vall d’Hebron, Barcelona, Spain. The study sample comprised 30 HIV-positive participants aged ≥ 18 years on the EFV/FTC/TDF regimen with HIV-RNA < 50 copies/mL for at least 6 months (blips were allowed), without other comorbidities or concomitant medications. Exclusion criteria included prior virologic failure (two consecutive HIV-RNA > 50 copies/mL), any acute or chronic disease that could interfere with the lipidomics analysis, alcohol or other drug abuse, body mass index (BMI) > 25, or use of drugs that could affect lipid metabolism at baseline or throughout the study, such as statins/fibrates, antidiabetic drugs, or steroids. Additionally, specific dietary or exercise interventions were not recommended during the trial to avoid any possible influence on the metabolic parameters. Patients were randomized (1:1) to continue their current regimen or to switch to RPV/FTC/TDF for 24 weeks (Figure 1). The study was approved by the ethics committee of the Hospital Vall d’Hebron and the Agencia Española del Medicamento y Productos Sanitarios (AEMPS). All participants gave their written informed consent in accordance with the Declaration of Helsinki. 

### 2.2. Demographic Characteristics and Conventional Biochemical Parameters

At baseline, the following parameters were recorded: demographics (age, sex, BMI, smoking and alcohol habits, physical activity), HIV-related factors (risk factor for HIV, time since diagnosis, time on ART, prior treatments, nadir CD4^+^ cell count, maximum HIV-RNA), physical examination (blood pressure, heart rate, temperature), concomitant medications, and pregnancy test if women were of childbearing potential. Blood tests included CD4^+^ and CD8^+^ cell counts, HIV-RNA, liver parameters, and conventional metabolic assessments, which included lipids (total cholesterol [TC], high-density lipoprotein cholesterol [HDL-C], low-density lipoprotein cholesterol [LDL-C], triglycerides [TG], apolipoprotein A [apoA] and apolipoprotein B [apoB]), fasting glucose, and insulin together with lipidomic profiling. Visits with blood tests were repeated at 12 and 24 weeks to perform conventional biochemical analysis (CBA) and lipidomic profiling. 

### 2.3. Lipidomics

Plasma lipidomic profiling was performed by OWL (http://www.owlmetabolomics.com/) using multiple ultra-high-performance liquid chromatography-mass spectrometry (UHPLC-MS) platforms for extensive coverage of the plasma lipidome [22]. Lipidome extraction was accomplished by fractionating the plasma samples into pools of species with similar physicochemical properties, using appropriate combinations of organic solvents, and the lipidome was analyzed following an untargeted procedure as previously described [23]. The coverage of the methanol extract platform (platform 1) included fatty acyls, bile acids (BA), steroids (ST), and lysoglycerophospholipids profiling, whereas the chloroform/methanol extract (platform 2) included glycerolipids, cholesteryl esters (ChoE), sphingolipids, and glycerophospholipids profiling. For each analytical platform, two different types of quality control (QC) plasma samples were used to assess the data quality. Data obtained were preprocessed using the TargetLynx application manager for MassLynx 4.1 software (Waters Corp., Milford, MA, USA). Normalization factors were calculated for each metabolite by dividing their intensities in each sample by the recorded intensity of an appropriate internal standard in that same sample. Any remaining sample injection variable response zero values in the corrected dataset were replaced with missing values before averaging to form the final dataset, which was used for the study sample statistical analyses [22,23]. Lipid classification followed the comprehensive classification system proposed by Fahy et al. [24] under the leadership of the International Lipid Classification and Nomenclature Committee (ILCNC) as expressed in the LIPID MAPS initiative (LIPID Metabolites and Pathways Strategy, Cambridge, UK; http://www.lipidmaps.org).

### 2.4. OWLiver^®^ Care and OWLiver^®^ Test

Two serum-based, BMI-dependent lipidomic tests were performed to estimate the presence of non-alcoholic fatty liver (NAFL) or non-alcoholic steatohepatitis (NASH) in this study cohort. OWLiver^®^ Care and OWLiver^®^ tests were used to estimate the presence of NAFL or NASH. The OWLiver^®^ Care test discriminates between normal liver condition and NAFLD, and the OWLiver^®^ test discriminates NAFL from the more serious NASH. These two noninvasive tests have been validated in clinical practice using blind-histology of HIV-uninfected subjects as a reference standard [25,26]. However, the results obtained in this study should be considered as a preliminary estimation because the efficacy of these tests has not been previously demonstrated in plasma samples or applied to PLWH. 

### 2.5. Statistical Analyses

For clinical and demographic data, categorical variables are described by number (percentage) and quantitative variables are described as medians (interquartile range). Qualitative variables were analysed using the χ^2^ test or Fisher’s exact test when necessary. Comparisons between groups were performed with non-parametric Mann-Whitney tests for unpaired samples and Wilcoxon t-test for paired samples.

For plasma lipidome data, the Shapiro-Wilk test was used for testing the normality of data and multivariate unsupervised principal components analysis (PCA) and supervised orthogonal partial least-squares to latent structures (OPLS) were used to reduce the dimensionality of the complex dataset and to identify the metabolites contributing to the clustering observed in the PCA plots to discriminate between groups, respectively. Volcano plot analysis was performed as an effective and easy-to-interpret graphing method that summarizes both fold-change and t-test criteria. Metabolites were evaluated by calculating group percentage changes and unpaired Student’s t-test *p*-value (normal distribution) or Wilcoxon signed-rank test (non-normal distribution) as well as Mann-Whitney tests (non-normal distribution) for the comparisons included in the study [22,27,28]. Pearson correlation coefficients and the corresponding *p* values were calculated to assess the associations between lipid metabolites and changes in lipid biomarkers used in clinical practice (TC, HDL, LDL, TG, apoA, and apoB).

Statistical analyses were performed using IBM SPSS statistics for Windows (version 20.0, Armonk, NY, USA: IBM Corp) and the R software computing environment (https://www.r-project.org/). PCA and OPLS multivariate data analysis were performed using the SIMCA-P1 software package (version 12.0.1; Umetrics, Umea, Sweden). The graphical representations are based on both the graphical environment of R, using a Shiny-based web application (OWL Stat App) and GraphPad Prism software (version 5.0, GraphPad Inc., San Diego, CA, USA). The results were considered significant at *p* < 0.05.

## 3. Results

### 3.1. Patient Characteristics

Thirty patients receiving stable EFV/FTC/TDF therapy were included in the study and were randomized to switch treatment to RPV co-formulated with FTC/TDF or to maintain the same regimen (Figure 1). A total of 29 patients completed the study and one patient was excluded due to a detectable viral load (HIV-RNA > 50 copies/mL) at baseline (protocol violation). There were no significant differences in baseline characteristics between groups, as described in Table 1. 

### 3.2. Conventional Biochemical Parameters 

**Figure 2:**
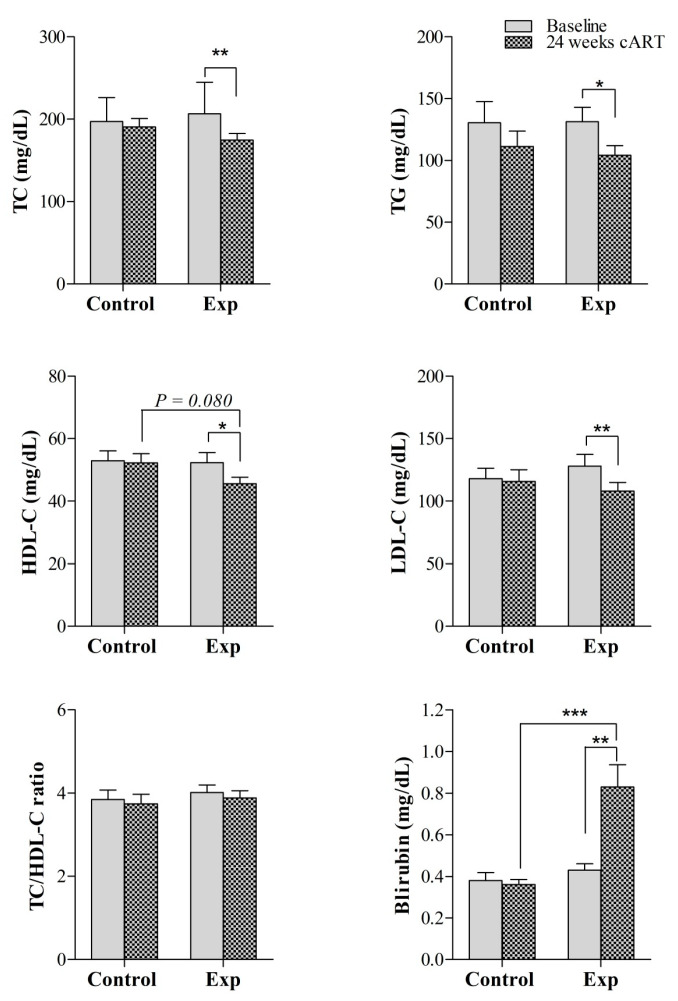
Evaluation of the effect of switching to RPV versus maintaining EFV both co-formulated with FTC/TDF on conventional clinical parameters at baseline and at 24 weeks of follow-up. Data are presented as the mean ± SEM. *p-value < 0.05, **p-value < 0.01, and *** *p*-value < 0.001. Abbreviations: Exp, experimental group; RPV, rilpivirine; TC, total cholesterol; TG, total triglycerides; LDL-c: low-density lipoprotein cholesterol; HDL-c: high-density lipoprotein cholesterol.

Regarding lipid parameters, significant reductions in TC, TG, apoA, and apoB plasma concentrations were observed in the experimental group after 24 weeks of ART compared to the corresponding baseline values (171 (158–195) mg/dL vs. 212 (89–237) mg/dL, *p* = 0.004, for TC; 105 (84–132) mg/dL vs. 127 (107–141) mg/dL, *p* = 0.039, for TG; 144 (131–150) mg/ vs. 149 (140–169) mg/dL, *p* = 0.021, for apoA; and 78 (68–920) mg/dL vs. 94 (84–107) mg/dL, *p* = 0.004, for apoB) (Figure 2 and Table S1). However, the reduction in these lipid parameters was not significant when compared to the levels in the control group (Figure 2 and Table S1). The TC/HDL-C ratio non-significantly decreased from baseline to a similar extent in both the control and experimental groups. Insulin did not differ during the 24 weeks of follow-up in either of the groups or when comparisons were performed at the end of the study between the experimental and control groups (Table S1). However, glucose concentrations were reduced after 24 weeks compared to baseline values in the experimental group (86 (80–95) mg/dL vs. 93 (83–100) mg/dL, *p* = 0.043), whereas no changes were observed in the control group (Table S2). Additionally, circulating total bilirubin was significantly increased in the experimental group during ART therapy (0.8 (0.5–0.9) mg/dL vs. 0.4 (0.3–0.5) mg/dL, *p* = 0.002) and the plasma concentrations at 24 weeks of follow-up were significantly higher than those in the control group (0.8 (0.5–0.9) mg/dL in the experimental group vs. 0.3 (0.3–0.4) mg/dL in the control group, *p* < 0.001) (Figure 2). 

### 3.3. Using Efavirenz or Rilpivirine in ART has a Different Effect on Lipid Metabolism

**Figure 3:**
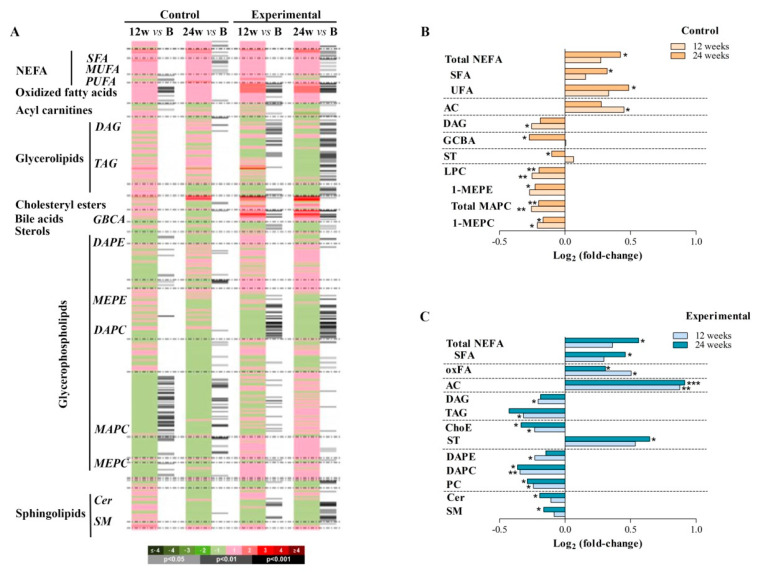
Effect of EFV and RPV on lipid metabolism. (**A**) Heatmap representing individual metabolic features obtained from the comparisons between the groups at 12 and 24 weeks after ART randomization. **(B, C)** Highlighted metabolite changes from the heatmap found at 12 and 24 weeks compared to the corresponding baseline values in patients on EFV (**B**) and RPV **(C)** in the ART. Heatmap colour codes for log2 (fold change) and paired Student’s t-test *p*-values are indicated on the bottom of the figure. Abbreviations: AC, acyl carnitines; B, baseline; Cer, ceramides; ChoE, cholesteryl esters; DAG, diacylglycerol; DAPC, diacylglycerophosphocholine; DAPE, diacylglycerophosphoethanolamine; GCBA, glycine-conjugated bile acid; LPC, lysophosphatidylcholine; MAPC, monoacylglycerophosphocholine; MEPC, monoetherglycerophosphocholine; MEPE, 1-monoetherglycerophosphoethanolamine; MUFA, monounsaturated fatty acid; NEFA, non-esterified fatty acid; oxFA, oxidized fatty acid; PC, phosphatidylcholine; PUFA, polyunsaturated fatty acid; SFA, saturated fatty acid; SM, sphingomyelin; ST, total steroid; TAG, triacylglycerol; UFA, unsaturated fatty acid.

A total of 366 metabolic features were detected using UHPLC-MS in the analysed plasma samples and were included in the subsequent univariate and multivariate data analyses. First, we examined the impact of using EFV (control) or RPV (experimental) on plasma lipids by comparing the lipid profiles at both 12 and 24 weeks to baseline values, in each group (Figure 3A). Although the control group had already been taking EFV for 6 years before enrolment, at 12 weeks of randomization, surprisingly there were significant increases in plasma concentrations of some acyl carnitines (ACs) but also decreased concentrations of saturated diacylglycerols (DAGs). However, the main metabolite class altered in this control group was the glycerophospholipids, including monoacylglycerophosphocholines (MAPC) and monoetherglycerophosphocholine (MEPC), which were found to be significantly decreased at both 12 and 24 weeks compared with the corresponding baseline values. Additionally, whereas non-esterified fatty acids (NEFAs), including saturated fatty acids (SFAs) and unsaturated fatty acids (UFAs), were significantly increased, total steroids (STs) and glycine-conjugated bile acids (GCBAs) were found to be significantly decreased at 24 weeks of follow-up compared to the corresponding baseline values (Figure 3B, Table S2). Thus, these results confirmed that continuous administration of the EFV/FTC/TDF regimen affects lipid metabolism regardless of the time of EFV exposure.

On the other hand, patients on RPV exhibited a prompt significant decrease in triacylglycerol (TAG), diacylglycerol (DAG), and cholesteryl esters (ChoEs). This experimental group of patients also exhibited a significant decrease in several glycerophospholipids at 12 weeks of randomization which remained significantly lower after 24 weeks compared to the respective baseline values. Similar to what was found for patients on EFV, ACs and the complete profile of NEFAs were significantly increased at both 12 and 24 weeks of ART compared to the respective baseline values. Contrary to what was previously observed in patients on EFV, patients on RPV showed increased values of some STs at the end of the study. Additionally, this experimental group revealed significantly decreased values of sphingolipids, including ceramides (Cers) and sphingomyelins (SMs), after 24 weeks (Figure 3C, Table S3).

### 3.4. Storage Lipids Decrease when Using RPV in Co-Formulated TDF/FTC ART Therapy 

**Figure 4:**
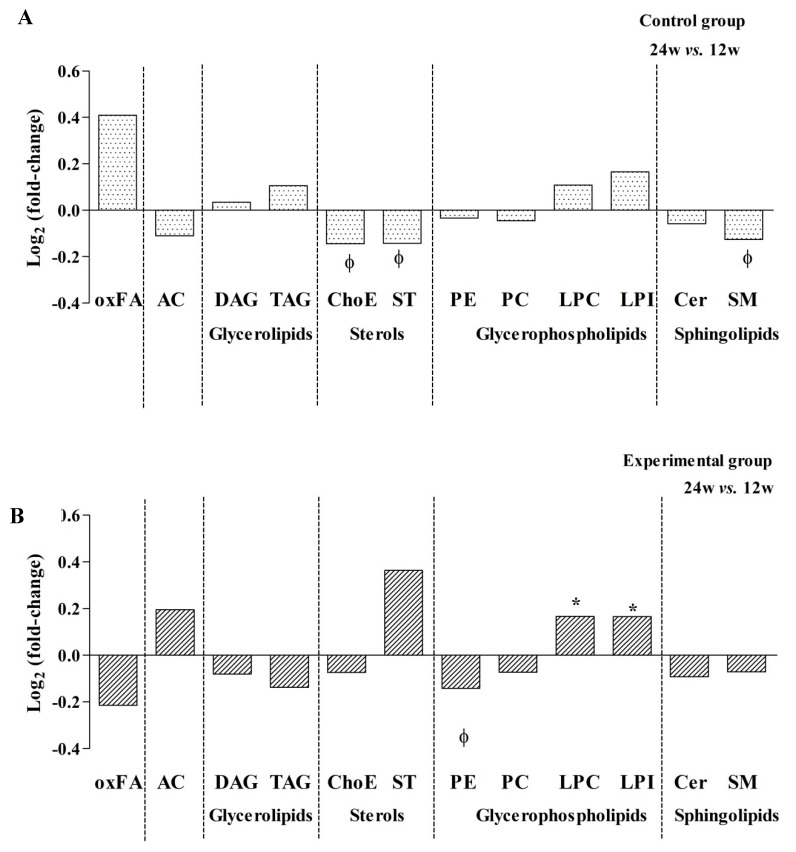
Effect of EFV and RPV on lipid metabolism. (**A**) Relevant metabolite changes grouped by chemical classes related to EFV or (**B**) RPV regimen. Data are expressed as log_2_ (fold change) and values between 24 and 12 weeks were compared using paired Student’s t-test *p*-values. *p values < 0.05 were considered significant and ^φ^*p* values >0.05 but <0.10 were considered relevant in the results interpretation. Abbreviations: AC, acyl carnitine; Cer, ceramide; ChoE, cholesteryl ester; DAG, diacylglycerols; LPC, lysophosphatidylcholine; LPI, lysophosphatidylinositol; oxFA, oxidized fatty acid; PC, phosphatidylcholine; PE, phosphatidylethanolamine; SM, sphingomyelins; ST, total steroid; TAG, triacylglycerol.

Second, we investigated the possible longer-term effects on lipid metabolism by comparing plasma samples obtained at 24 weeks to those obtained at week 12 of ART in each group of patients. Our results showed different end-point effects on lipid metabolism according to ART therapy. On the one hand, the control group showed a general decrease in sterols and sphingolipids during the study follow-up (Figure 4A). When individual compounds were evaluated, 20 metabolites were significantly altered. The most relevant changes were a decrease in some classes of ChoEs, SMs, and 1-ether, 2-acylglycerophosphocholine (MEMAPC) compounds. MEMAPCs are phosphatidylcholines (PCs) with one fatty alcohol of varying lengths and saturation profiles attached in the C-1 position (Table S4**)**. On the other hand, the experimental group revealed a significant decrease of phosphatidylethanolamines (PEs) but also an increase in lysophosphatidylcholines (LPCs) and lysophosphatidylinositols (LPIs) (Figure 4B). In this context, 29 metabolites were significantly altered due to ART. The main changes were due to a decrease in some classes of diacylglycerophosphocholines (DAPCs), which are PCs with one saturated fatty acid of varying lengths and saturation profiles attached in the C-1 position, as well as an increase in some MAPC lipid species, including monoacylglycerophosphoinositol (MAPI) and monoetherglycerophosphocholine (MEPC) (Table S4).

Interestingly, patients switching from EFV to RPV showed a decrease in the levels of oxidized fatty acids (oxFAs) as well as glycerolipids, suggesting that RPV could have less impact on TAG levels, which jointly contributed to a decrease in ChoE and contributed to a reduction in the amount of storage lipids (SL) (-0.137 log_2_ (fold-change) in the experimental group vs. 0.059 log_2_ (fold-change) in the control group). 

### 3.5. Effect of switching from EFV to RPV: decreased PCs but increased LPCs and ACs 

**Figure 5:**
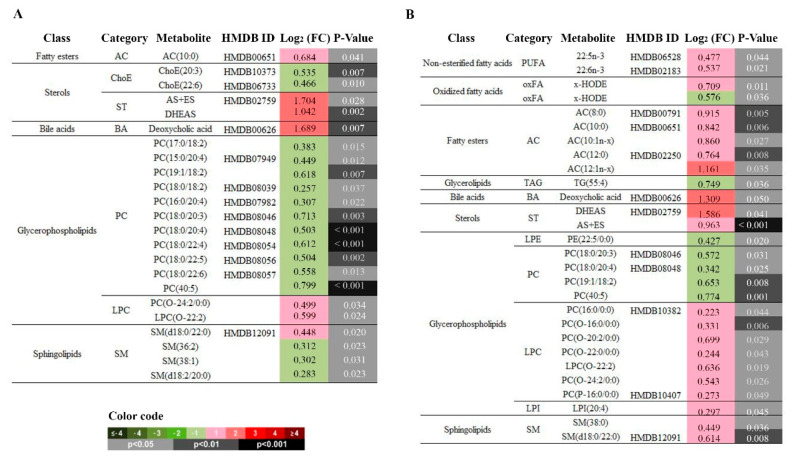
Impact of switching from EFV to RPV on the lipidomic profile. Relevant lipidomic changes grouped by chemical classes at (**A**) 12 weeks and at (**B**) 24 weeks of follow-up. Heatmap colour codes for log_2_ (fold-change) and unpaired Student’s t-test *p*-values are indicated at the bottom of the figure. Abbreviations: AC, acyl carnitine; AS+ES (androsterone sulfate + etiocholanolone sulfate); BA, bile acid; Cer, ceramides; ChoE, cholesteryl esters; DAG, diacylglycerol; DHEAS, dehydroepianosterone sulfate; FC, fold change; HMDB, Human Metabolome Database; HODE, hydroxy-octadecadenoic acid; LPC, lysophosphatidylcholine; LPE, lysophosphatidylethanolamine; LPI, lysophosphatidylinositol; oxFA, oxidized fatty acid; PC, phosphatidylcholine; PE, Phosphatidylethanolamine; PUFA, Polyunsaturated fatty acid; SL, storage lipid; SM, sphingomyelin; ST, total steroid; TAG, triacylglycerol.

Finally, we wanted to evaluate the effect of switching from EFV to RPV on lipid metabolism at 12 and 24 weeks of follow-up. From a total of 366 metabolites detected in plasma samples, 23 metabolites and 28 metabolites were significantly altered in the experimental group compared to the levels in the control group at 12 and 24 weeks, respectively. At both 12 and 24 weeks, the most remarkable changes were a decrease in several DAPCs (PCs) (i.e., log_2_(FC) = 0.503, *p* < 0.001, at 12 weeks and log_2_(FC) = 0.342, *p* = 0.025, at 24 weeks for PC(18:0/20:4)) and an increase in some MEPCs (LPCs) (i.e., log_2_(FC) = 0.599, *p* = 0.024 at 12 weeks and log_2_(FC) = 0.636, *p* = 0.0019 at 24 weeks for LPC(O-22:0)) when using RPV instead of EFV (Figure 5A). At 24 weeks, switching from EFV to RPV was also associated with a significant increase in some ACs (global log_2_(FC) = 0.616, *p* = 0.01) (Figure 5B). Although not significant, a global decrease in the TAG content and storage lipids (TAG+ChoE) was observed in patients who switched to RPV compared to those who continued taking EFV. 

### 3.6. Good Correlation between Lipidomics and Conventional Laboratory Measurements

To validate the lipidomic changes, we performed correlation analysis between lipid parameters obtained by conventional laboratory measurements and the 366 metabolites detected by UHPLC-MS. A positive correlation was observed between HDL-C and MEPCs (LPC), whereas LDL-C exhibited a negative association with DAPC (PCs) in patients who switched to RPV. The positive correlation between apoA and glycine and taurine-conjugated bile acids was also notable in patients who switched from EFV to RPV at 24 weeks. Total TGs and apoB showed a positive correlation with most of the TAG detected and the relationship of SM content in plasma with total cholesterol was stronger in patients who continued with EFV at 24 weeks (Figures S1 and S2). 

### 3.7. Evaluation of non-Alcoholic Fatty Liver Disease using Plasma Samples 

First, conventional biochemical analyses showed a significant reduction in circulating alkaline phosphatase (ALP) and gamma-glutamyl transferase (GGT) concentrations during follow-up within the experimental group, and GGT was also significantly reduced compared to the level in the control group at 24 weeks (Figure S3).

Second, the OWLiver^®^ Care test was performed on the plasma samples to estimate the possible presence and evolution of NAFLD in each group of patients. It could not be performed in 2 participants in each group. The test estimated that nine patients in the EFV group and 11 patients in the RPV group exhibited fatty liver diseases after 24 weeks. Two patients maintaining the use of EFV with apparently normal liver function at baseline developed NASH after 24 weeks and three patients switched to RPV who exhibited steatosis at baseline progressed to NASH after 24 weeks of follow-up, respectively (Figure S3).

Third, as mentioned above, lipidomics revealed a global decrease in the amount of SL when EFV was changed to RPV in the co-formulated TDF/FTC ART therapy. Notably, DAG, TAG, and Cer were specifically decreased in the experimental group, contributing to a reduction in the amount of lipid storage organelles (lipid droplets) associated with excessive accumulation of lipids in ectopic tissue (Figure 4, Figure S3).

### 3.8. Effectiveness and Adverse Events

No differences in CD4^+^ T-cell count between the ART therapies were observed at the end of the study (Table S1). One patient from the control group developed virologic failure due to non-adherence. 

There were no suspected severe adverse reactions related to the study drugs. One patient from the control group had a severe adverse event not related to the study medication (dental abscess that required surgical drainage). 

## 4. Discussion

Guidelines recommended EFV combined with two NRTIs as a preferred regimen for ART-naïve PLWH for many years, and many patients are still receiving this drug. For that reason, the effect of continuing EFV or switching to RPV, which seems to improve the lipid profile in PLWH, has been previously evaluated using conventional biochemical parameters [13,14,15,16,17]. However, lipidomics analysis demonstrated that the human plasma lipidome comprises several individual species that are overlooked using conventional biochemical techniques. Lipids, due to their chemical and structural diversity, have diverse metabolic functions, including metabolic, inflammatory, and immune response functions [19,20,29], and imbalances in lipid metabolism and signalling are linked to the pathology of various diseases, including CVD and NAFLD [18,30,31,32,33,34]. Thus, in this study, we performed plasma lipidome screening to contrast the underlying changes in lipid homeostasis associated with switching from EFV to RPV in the ART regimen co-formulated with TDF/FTC. To our knowledge, this is the first study that has identified lipid species that may be related to a better metabolic profile with the use of RPV rather than EFV.

First, we used conventional laboratory measurements to identify biochemical changes associated with continuing on EFV or switching to RPV in PLWH. We identified significant decreases in circulating TC, LDL-C, and TG, which are risk factors for CVD, after switching from EFV to RPV. A significant decrease in HDL-C was also observed during treatment within the RPV regimen, but the decrease in the TC to HDL-C ratio, a key lipoprotein predictor of future CVD, was not statistically significant; in contrast, circulating bilirubin levels were significantly increased. The TC/HDL-C ratio was formerly considered a risk indicator with greater predictive value than isolated parameters used independently [35], but some studies have revealed that errors occur when using the conventional TC/HDL-C ratio to estimate the risk of vascular disease [36]. On the other hand, a significant negative relationship between serum bilirubin levels and CVD, resulting in decreased circulating cholesterol and triacylglycerol concentrations, was described in PLWH [37]. In fact, higher serum total bilirubin could have antioxidant properties (through the inhibition of low-density lipoprotein oxidation), anti-inflammatory effects (mediated in part by reducing oxidative stress), and antiatherogenic properties that contribute to reduce CVD risk and CVD death [38]. Thus, overall, our biochemical results corroborated previous findings that support that switching from EFV to RPV results in favourable changes in the lipid profile, which may induce the activation of mechanistic pathways towards the reduction in CVD risk in PLWH [15,16]. 

We then characterized the plasma lipidome and identified alterations in several lipid classes that perfectly correlated with changes in lipid biomarkers used in conventional clinical practice. The most notable lipid alterations in participants who switched from EFV to RPV were observed in glycerolipids, glycerophospholipids, and fatty esters. In particular, treatment with RPV resulted in an early decrease in DAPC, which are PCs, and a significant increase in some MEPC, which are LPCs. Phosphatidylcholine, one of most abundant phospholipids in the cell membrane, is rapidly metabolized by the phospholipase A2 (PLA2) enzyme to LPC and a free fatty acid and can be converted back to a PC by the enzyme lysophosphatidylcholine acyltransferase (LPCAT) in the presence of acyl-CoA [39,40]. Thus, our results may indicate that the cleavage of PC via the action of PLA2 is enhanced in the circulation of patients on the RPV regimen. LPC is a major component of oxidized LDL (oxLDL) with both pro- and anti-inflammatory properties. Previously, LPCs were identified as a group of pro-inflammatory and pro-atherogenic lipids that are critically involved in the pathogenesis of atherosclerosis and other inflammatory diseases [39,40]. Increased levels of LPC in ART-naïve and ART-treated HIV patients have been previously related to monocyte and immune activation and associated with comorbid disease pathogenesis [19,20]. However, findings from recent clinical lipidomics studies are controversial and have suggested that LPC could stimulate HDL-mediated cholesterol efflux, inhibit cholesterol biosynthesis, enhance the extracellular expression of antioxidant enzymes, and attenuate the oxidation of LDL–C [40]. In fact, LPC exhibits anti-inflammatory and anti-atherogenic properties based on its fatty acid composition [30,31]. Increased levels of saturated fatty acid (SaFA)-containing LPCs tend to have pro-atherogenic properties, whereas increased polyunsaturated fatty acid (PUFA)-containing LPCs seem to be related to a reduced risk of CVD and mortality in HIV-infected individuals [19]. Interestingly, our patients on the RPV regimen showed a significant increase in PUFA content and jointly, the positive correlation between plasma HDL-C concentrations and MEPC levels may indicate an improved CVD prognosis compared to that of patients taking EFV. Moreover, PUFA-containing phospholipids were negatively associated with liver fat content, becoming a useful indicator of NAFLD in a diagnostic model combined with specific TGs [33]. According to these data, the results from OWLiver^®^ Care revealed that no patient on the RPV regimen with an apparent normal liver at baseline developed NASH after 24 weeks. Unfortunately, no images of the measure of carotid intimal-medial thickness or measures of hepatic function were available for these patients to accurately corroborate our findings. It is important to remark that OWLiver^®^ Care is a noninvasive test developed by OWL based on serum samples and has been validated in clinical practice and compared directly to the results of invasive liver biopsies in HIV-uninfected patients [25,26]. In this study, the test was performed in plasma samples and applied to HIV-infected patients; thus, our results should be carefully interpreted and only used for research purposes until more data are available. 

The global amount of storage lipids (especially DAG, TAG, and Cer) was decreased with RPV, which can contribute to reducing the repository of specific lipotoxic lipids in multiple tissues. An excessive accumulation of storage lipids in the liver represents a major risk factor for the development of NAFLD [32]. At a cellular level, DAG can be esterified to TAG and the continuous formation of TAG leads to the formation of specialized lipid storage organelles, commonly known as lipid droplets (LDs). Lipolysis of TAG also results in the formation of the lipid intermediate DAG, which can be further hydrolysed to provide fatty acids (FAs) for energy production, membrane lipid synthesis, or very-low-density lipoprotein (VLDL) synthesis in the liver. Excessive supply of FAs and subsequent uptake into different tissues can contribute to an increase in cellular Cer. Moreover, some ACs were elevated in both groups of patients. ACs are the rate-limiting substrate in mitochondrial β-oxidation, the principal oxidative pathway for oxidation of FAs. Increased serum AC has been previously reported in patients with diabetes and metabolic, cardiovascular, and mitochondrial diseases as well as in HIV patients receiving PI-based ART [29]. Therefore, elevated levels of AC in the plasma of both groups in this study could be related to the ART regimen and may be indicative of mitochondrial β-oxidation impairment. In fact, the OWLiver^®^ Care test estimated that nine patients treated with the EFV regimen and eleven treated with the RPV regimen showed fatty liver diseases after 24 weeks of follow-up. However, it is important to remark that more than half of the included patients, with normal BMI and no known comorbidities, already presented steatosis before randomization (7 in the arm continuing with EFV and 9 in the arm switching to RPV). 

As mentioned above, the plasma lipidome signature of patients treated with RPV revealed a significant decrease in Cer and SM values. Cers are sphingolipids that contain a sphingosine backbone attached to a FA, and SM is a ceramide metabolite and precursor. Cer and SM are both independent risk factors for atherosclerosis [41]. Inhibition of Cer and SM biosynthesis was associated with improved plasma lipids and lipoproteins, related to a significant reduction in the overall CVD risk by regulating HDL-C functionality and linked to diet-induced NAFLD due to its key role in hepatic triglyceride accumulation and inflammation, as well as to hepatic oxidative stress, fibrosis, and apoptosis [34]. Interestingly, a simple Cer- and PC-based risk score has been recently developed to efficiently predict residual CVD event risk in patients with coronary artery disease [42]. As mentioned above, patients on RPV showed decreased values of PCs, which corroborate this synergistic effect of Cer levels in the prevention of CVD and NAFLD-associated atherosclerosis.

Remarkably, our data also showed that switching to RPV increased the levels of total steroids. Steroid hormones are cholesterol-derived molecules that are often used in the setting of inflammation to attenuate the physiologic response. Steroids work to delay hypersensitivity, inhibit antibody-dependent cytotoxicity, inhibit the release of lysosomal enzymes, and decrease cytotoxic T cells, the migration of neutrophils, and some pro-inflammatory interleukins [43]. In fact, correlation analysis identified a negative association between sulfated steroids and IFN-α and IL-6, markers of innate immune activation, in HIV patients receiving PI-based ART [29]. Local and systemic actions of steroids provide novel therapeutic avenues to improve the outcome of CVD and NAFLD, although prolonged and excessive exposure to steroids is also related to metabolic complications. In the aorta, steroids may have both protective effects, preventing neointimal proliferation, and adverse effects, preventing angiogenesis and promoting perivascular inflammation and myocardial fibrosis [44]. In the liver, steroids are related to the stimulation of both hepatic influx (via de novo lipogenesis) and efflux of lipids (via VLDL production) [45]. In the present study, the increase in steroids in the RPV group was considered a protective mechanistic response rather than an adverse incident, as the concentration observed was within the normal physiological response range and was not associated with an excess of endogenous production or prolonged hazardous exposure.

Several limitations to our study should be noted. The first is the small number of patients included, together with the risk of confounding variables. However, we selected stable patients without other comorbidities or drugs that could have an impact on the metabolic profile to reduce heterogeneity. The only change was the substitution of EFV by RPV in half of the patients. Second, the included patients were on stable ART with EFV/FTC/TDF for more than 6 years. This could result in selection bias as patients with metabolic toxicity due to EFV would probably have modified their ART previously. Furthermore, dyslipidaemia was an exclusion criterion. Therefore, the results might have underestimated real lipid differences between the EFV and RPV groups. Third, patients received TDF/FTC as the NRTI backbone, whereas there is now widespread use of TAF instead of TDF. TAF has a different lipid profile than TDF, which could prevent the generalization of our results to patients receiving RPV with TAF. However, recent concerns have arisen regarding the risk of weight increase with TAF [7]. On the other hand, as mentioned above, OWLiver^®^ Care and OWLiver^®^ tests were based on serum samples from HIV-uninfected patients and their efficacy has not been demonstrated in plasma samples; thus, these results should be carefully considered when applied to HIV patient plasma samples. 

In summary, lipidomic profiling identified different alterations in the plasma lipidome of HIV patients on stable ART who switched from EFV to RPV from those who continued on EFV in the ART regimen co-formulated with TDF/FTC. The distinct lipid classes significantly altered in patients who switched to RPV suggests that the use of this drug in the ART regimen could have a beneficial effect on the lipid profile compared to the effects of the EFV regimen. Concretely, the RPV may prevent excessive LD accumulation in multiple tissues, which represents a major risk for CVD and NAFLD-associated atherosclerosis in PLWH. In fact, biochemical parameters corroborated with our lipidomic results, suggesting an improved global lipid profile in patients taking RPV. Further causative exploration of these results is required to determine the role of specific drugs.

## Figures and Tables

**Table 1:** Clinical and epidemiological baseline characteristics of the study cohort.

Title	Experimental (n = 15)	Control (n = 14)	Total (n = 29)	*p*- value
Age, years	45 (36–52)	44 (35–45)	44 (36–48)	0.373
Male	12 (80)	13 (87)	25 (83)	1
BMI, kg/m^2^	23.9 (21.5–24.8)	22.7 (20.9–24.4]	23.8 (21.5–24.6]	0.361
Race/ethnicity				0.597
Hispanic	1 (7)	3 (20)	4 (13)	
Caucasian	13 (87)	12 (80)	25 (83)	
African	1 (7)	0 (0)	1 (3)	
Current smoker	6 (40)	5 (33)	11 (37)	0.705
HIV infection, years	10 (7–13)	9 (7–16)	10 (7–15)	0.604
HIV risk factor				1
Heterosexual	4 (27)	4 (27)	8 (27)	
MSM	10 (67)	9 (60)	19 (63)	
Injection drug abuse	0 (0)	1 (7)	1 (3)	
Other	1 (7)	1 (7)	1 (3)	
Prior AIDS	3 (20)	1 (7)	4 (13)	0.598
Time on ART, years	7 (4–13)	8 (5–16)	7 (5–14)	0.330
Time on EFV/FTC/TDF, years	6 (4–8)	6 (4–9)	6 (4–8)	0.756
Nadir CD4^+^ count, cells/µL	224 (109–400)	240 (168–365)	236 (139–390)	0.576
Current CD4^+^ count, cells/µL	730 (560–950)	740 (540–1000)	740 (540–950)	0.678
Current CD8^+^ count, cells/µL	675 (510–760)	630 (490–830)	660 (510–820)	0.861
Lipid parameters				
TC, mg/dL	214 (189–240)	200 (184–226)	207 (185–234)	0.175
LDL-c, mg/dL	137 (122–157)	126 (93–147)	133 (96–151)	0.141
HDL-c, mg/dL	58 (40–64)	54 (41–64)	57 (41–64)	0.927
Triglycerides, mg/dL	122 (103–141]	115 (87–166)	117 (100–152)	0.646
apoA, g/dL	149 (140–178]	150 (128–172)	149 (133–172)	0.468
apoB, g/dL	94 (83–108]	88 (70–100)	91 (74–103)	0.340
Other laboratory parameters				
ALT, UI/L	29 (22–38]	23 (20–31)	23 (21–32)	0.197
ALP, UI/L	116 (80–135]	94.50 (84–134)	98 (80–134)	0.854
AST, UI/L	22 (20–32]	24 (20–27)	23 (20–28)	0.729
Bilirubin, mg/dL	0.42 (0.35–0.52]	0.35 (0.27–0.39)	0.38 (0.33–0.47)	0.135
GGT, UI/L	53 (37–61]	36 (28–66)	45 (31–63)	0.141
Glucose, mg/dL	92 (83–100]	89 (83–90)	90 (83–99)	0.596
Insulin, mU/L	15.0 (10.4–17.8)	11.6 (7.6–28.1)	14.2 (9.3–22.7)	0.604
Total protein, g/dL	7.20 (7.00–7.60)	7.20 (7.00–7.50)	7.20 (7.00–7.55)	0.854

Data are presented as n (%) or median (interquartile range). Categorical data were compared by means of a χ^2^ test, whereas continuous data were compared using nonparametric Mann-Whitney test. BMI: Body Mass Index; MSM: men who have sex with men; ART: antiretroviral treatment; EFV: efavirenz; FTC: emtricitabine; TDF: tenofovir disoproxil fumarate; TC: total cholesterol; LDL-c: low-density lipoprotein cholesterol; HDL-c: high-density lipoprotein cholesterol; apoA: Apolipoprotein A; apoB: Apolipoprotein B; ALT: alanine aminotransferase; ALP: Alkaline phosphatase; AST: Aspartate aminotransferase; GGT: Gama-glutamyl transferase.

## Supplementary Materials

The following are available online at https://www.mdpi.com/2077-0383/9/5/1246/s1, Table S1: Evaluation of the ART effect for the most relevant clinical parameters (*n* = 29; 24 weeks of follow-up), Table S2: Heatmap representing individual metabolic compounds significantly altered at 12 weeks or 24 weeks of receiving EFV/FTC/TDF compared to baseline values, Table S3: Heatmap representing individual metabolic compounds significantly altered at 12 weeks or 24 weeks of receiving RPV/FTC/TDF compared to baseline values, Table S4: Heatmap representing significantly altered metabolites obtained for the comparison between 24 weeks to 12 weeks in patients receiving EFV/FTC/TDF and RPV/FTC/TDF respectively, Figure S1: Heatmap representing Pearson’s correlations between lipid metabolites obtained by UHPLC-MS and lipid parameters conventionally used in clinical practice at 12 and 24 weeks compared to baseline (B) in patients taking EFV/FTC/TDF (1) or RPV/FTC/TDF (2) in the ART, Figure S2: Heatmap representing Pearson’s correlations between lipid metabolites obtained by UHPLC-MS and apolipoprotein A (apoA) and apolipoprotein B (apoB) obtained by conventional laboratory measurements at 12 and 24 weeks compared to baseline (B) in patients taking EFV/FTC/TDF (1) or RPV/FTC/TDF (2) in the ART, Figure S3: Effect of switching from EFV to RPV in the liver condition.
