# Supplementary material for: Lipidomics Reveals Reduced Inflammatory Lipid Species and Storage Lipids after Switching from EFV/FTC/TDF to RPV/FTC/TDF: A Randomized Open-Label Trial
Source: J Clin Med. 2020 Apr 25;9(5):1246. doi: 10.3390/jcm9051246 (PMC7288166; doi:10.3390/jcm9051246)
Supplement: Supplementary file 1 [file jcm-09-01246-s001.pdf]

Supplemental material for

Lipidomics reveals reduced inflammatory lipid species and storage lipids after switching from EFV/FTC/TDF to RPV/FTC/TDF: a randomized open-label trial

Adrian Curran[1,2, †,*], Anna Rull[3,†], Jordi Navarro[1,2], Judit Vidal-González[2,4], M Martin[2], Joaquin Burgos[1,2], Vicenç Falcó[1,2], Esteban Ribera[1,2], Ariadna Torrella[2], Bibiana Planas[2], Joaquim Peraire[3], Manuel Crespo[5]

1. Infectious Diseases Department, Hospital Universitari Vall d'Hebron, Barcelona, Spain.
2. Vall d'Hebron Institut de Recerca (VHIR), Barcelona, Spain.
3. Hospital Universitari de Tarragona Joan XXIII, IISPV, Universitat Rovira i Virgili, Tarragona, Spain.
4. Department of Internal Medicine - Hepatology, Hospital Universitari Vall d'Hebron (HUVH), Barcelona, Spain.
5. Internal Medicine Department. Complexo Hospitalario Universitario de Vigo; IIS Galicia Sur, Vigo, Spain.

[†] The first two authors contribute equally for this work.
[*]Author to whom correspondence should be addressed: **Adrian Curran** (acurran@vhebron.net)

Table S1. Evaluation of the ART effect for the most relevant clinical parameters (n =29; 24 weeks of follow-up).

Table S2. Heatmap representing individual metabolic compounds significantly altered at 12 weeks or 24 weeks of receiving EFV/FTC/TDF compared to baseline values.

Table S3. Heatmap representing individual metabolic compounds significantly altered at 12 weeks or 24 weeks of receiving RPV/FTC/TDF compared to baseline values.

Table S4. Heatmap representing significantly altered metabolites obtained for the comparison between 24 weeks to 12 weeks in patients receiving EFV/FTC/TDF and RPV/FTC/TDF respectively.

Figure S1. Heatmap representing Pearson's correlations between lipid metabolites obtained by UHPLC-MS and lipid parameters conventionally used in clinical practice at 12 and 24 weeks compared to baseline (B) in patients taking EFV/FTC/TDF (1) or RPV/FTC/TDF (2) in the ART.

Figure S2. Heatmap representing Pearson's correlations between lipid metabolites obtained by UHPLC-MS and apolipoprotein A (apoA) and apolipoprotein B (apoB) obtained by conventional laboratory measurements at 12 and 24 weeks compared to baseline (B) in patients taking EFV/FTC/TDF (1) or RPV/FTC/TDF (2) in the ART.

Figure S3. Effect of switching from EFV to RPV in the liver condition.

List of abbreviations

AC, acyl carnitines

ALP, alkaline phosphatase

ART, antiretroviral therapy

AS, androsterone sulfate

BA, Bile acids

BMI, body mass index

Cer, Ceramides

ChoE, Cholesteryl esters

DAG, diacylglycerols

DAPC, diacylglycerophosphocholine

DAPE, diacylglycerophosphoethanolamine

DHEAS, dehydroepianosterone sulfate

EFV, efavirenz

ES, etiocholanolone sulfate

FC, Fold change

FTC, emtricitabine

GCBA, glycine-conjugated bile acids

GGT, gamma-glutamyl transferase

HMDB, Human Metabolome Database

HODE, Hydroxy-octadecadenoic acids

LPC, lysophosphatidylcholines

LPE, lysophosphatidylethanolamines

LPI, lysophosphatidylinositols

MAPC, monoacylclycerophosphocholines

MAPE, Monoacylglycerophosphoethanolamine

MAPI, Monoacylglycerophosphoinositol

MEMAPC, 1-ether, 2-acylglycerophosphocholines

MEMAPE, 1-ether, 2-acylglycerophosphoethanolamine

MEPC, monoetherglycerophosphocholines

MEPE, 1-monoetherglycerophosphoethanolamine

MUFA, monounsaturated fatty acids

NEFA, non-esterified fatty acids

oxFA, oxidized fatty acids

PC, phosphatidylcholines

PE, Phosphatidylethanolamines

PUFA, Polyunsaturated fatty acids

SFA, saturated fatty acids

SM, sphingomyelins

SL, storage lipids

ST, total steroids

TAG, triacylglycerol

TDF, tenofovir

UFA, unsaturated fatty acids

UHPLC-MS, ultra-high performance liquid chromatography – mass spectrometry

Table S1. Evaluation of the ART effect for the most relevant clinical parameters (n =29; 24 weeks of follow-up).

	**Experimental (n=15)**		**P- value**	**Control (n=14)**		**P- value**	**P- value***
	**Baseline**	**End-Point**		**Baseline**	**End-Point**		
$CD4^+$ count, cells/ µL	770 [650 - 950]	710 [560 - 800]	0.326	740 [540 - 1000]	760 [580 - 990]	0.865	0.275
$CD8^+$ count, cells/ µL	690 [610 - 760]	620 [520 - 660]	*0.133*	630 [490 - 830]	660 [520 - 1070]	0.478	0.419
Lipid parameters							
Total Cholesterol, mg/dL	212 [189 - 237]	171[158 - 195]	**0.004**	200 [184 - 226]	185 [164 - 214]	0.363	**0.265**
LDL-cholesterol, mg/dL	137 [122 – 148]	107 [93 – 130]	**0.012**	126 [93 – 147]	119 [85 – 135]	0.683	**0.585**
HDL-cholesterol, mg/dL	57 [40 - 62]	47 [40 - 49]	**0.013**	54 [41 – 64]	52 [41 – 61]	0.802	***0.080***
Triglycerides, mg/dL	127 [107 – 141]	105 [84-132]	**0.039**	115[87-166]	98 [69 – 160]	0.221	**0.600**
Apolipoprotein A, g/dL	149 [140 – 169]	144 [131 – 150]	**0.021**	150 [128 – 172]	149 [126 – 162]	0.932	**0.197**
Apolipoprotein B, g/dL	94 [84 – 107]	78 [68 – 92]	**0.033**	88 [70 – 100]	86 [65 – 90]	0.173	**0.782**
Other Laboratory parameters							
Alanine transaminase (ALT), UI/L	28 [22 -37]	26 [23 – 36]	0.460	23 [20 – 31]	27 [18 – 30]	0.528	**0.284**
Alkaline phosphatase (ALP), UI/L	111 [80 -135]	94 [70 – 109]	**0.001**	95 [84 – 134]	95 [78 – 130]	0.330	**0.631**
Aspartate aminotransferase (AST), UI/L	22 [20 – 26]	24 [23 – 30]	0.182	24 [20 – 27]	23 [22 – 28]	0.777	0.254
Bilirubin, mg/dL	0.4 [0.3 – 0.5]	0.8 [0.5 – 0.9]	**0.002**	0.4 [0.3 – 0.4]	0.3 [0.3 – 0.4]	0.925	**<0.001**
Gama-glutamyl transferase (GGT), UI/L	52 [37 – 56]	28 [24 -28]	**0.002**	36 [28 – 66]	39 [30 – 74]	0.343	**0.036**
Glucose, mg/dL	93 [83 – 100]	86 [80 – 95]	**0.043**	89 [83 – 94]	93 [84 – 96]	0.925	0.393
Insulin (mU/L)	15.3 [11.7 – 17.8]	13.4 [6.6 – 16.5]	0.173	11.5 [7.6 – 28.1]	12.3 [9.6 – 14.1]	0.570	0.729
Total protein, g/dL	7.2 [7.0 – 7.5]	7.2 [6.7 – 7.2]	0.249	7.2 [7.0 – 7.5]	7.3 [6.9 – 7.4]	0.510	0.554

Data presented as median [interquartile range]. Differences between end-point (24 weeks of ART) and baseline values were compared using Wilcoxon t-test for paired samples (same group) (P-value). P-value* indicates differences at end-point (24 weeks of ART) between control and experimental group (unpaired t-test) (P values < 0.05 were considered significant and are highlighted in bold, whereas P values > 0.05 but < 0.15 were considered relevant for results interpretation and are italicized.

Table S2. Heatmap representing individual metabolic compounds significantly altered at 12 weeks or 24 weeks of receiving EFV/FTC/TDF compared to baseline values.

Metabolite	Metabolite Class	Individual notation	12 weeks *versus* baseline			24 weeks *versus* baseline		
			FC	log_2(FC)	P-value	FC	log_2(FC)	P-value
Total NEFA	Non-esterified fatty acids	Non-esterified fatty acids	1.209	0.274	**5.87E-01**	1.341	0.423	**3.15E-02**
NEFA_omega_6	Non-esterified fatty acids	NEFA omega 6	1.315	0.395	**3.71E-01**	1.488	0.574	**1.73E-02**
SFA	Non-esterified fatty acids	Saturated fatty acids	1.115	0.157	**7.75E-01**	1.250	0.322	**2.43E-02**
UFA	Non-esterified fatty acids	Unsaturated fatty acids	1.260	0.333	**5.44E-01**	1.401	0.487	**4.56E-02**
FFA.18.3	Non-esterified fatty acids		1.510	0.595	**4.70E-02**	1.523	0.607	**1.32E-02**
AC	Acylcarnitines	Acylcarnitines	1.367	0.451	**1.07E-02**	1.212	0.277	**2.02E-01**
DAG	Glycerolipids	Saturated Diacylglycerols	0.839	-0.253	**4.18E-02**	0.879	-0.187	**8.17E-02**
GCBA	Bile Acids	Glycine-conjugated bile acids	1.004	0.006	**8.39E-02**	0.828	-0.272	**2.71E-02**
ST	Sterols	Steroids	1.047	0.066	**8.70E-01**	0.933	-0.100	**3.56E-02**
LPC	Glycerophospholipids	Lysophosphatidylcholines	0.840	-0.251	**4.96E-03**	0.872	-0.198	**3.04E-03**
LPC + ChoE	Glycerophospholipids + ChoE		0.872	-0.198	**9.78E-03**	0.877	-0.190	**2.15E-03**
Lipids Membrane	Glycerophospholipids + DAG	PC+PE+LPC+LPE+PI+LPI+PS+PG+LPG(+DAG)	0.911	-0.135	**2.92E-02**	0.898	-0.155	**4.09E-02**
1-MEPE	Glycerophospholipids	1-Monoetherglycerophosphoethanolamine	0.829	-0.270	**9.92E-02**	0.855	-0.227	**1.83E-02**
MEPE.O_plasmanyles	Glycerophospholipids	1-Monoetherglycerophosphoethanolamine O_plasmanyles	0.835	-0.261	**1.20E-01**	0.813	-0.298	**6.82E-03**
MEPE.P_plasmenyles	Glycerophospholipids	1-Monoetherglycerophosphoethanolamine P_plasmenyles	0.832	-0.265	**1.01E-01**	0.864	-0.211	**2.94E-02**
Total MAPC	Glycerophospholipids	Monoacylglycerophosphocholine	0.838	-0.256	**3.25E-03**	0.870	-0.201	**4.42E-03**
1-MAPC	Glycerophospholipids	1-Monoacylglycerophosphocholine	0.832	-0.266	**2.18E-03**	0.861	-0.216	**3.90E-03**
2-MAPC	Glycerophospholipids	2-Monoacylglycerophosphocholine	0.843	-0.247	**4.55E-03**	0.874	-0.194	**4.76E-03**
1-MEPC	Glycerophospholipids	1-Monoetherglycerophosphocholine	0.864	-0.211	**4.90E-02**	0.891	-0.167	**3.90E-02**
MEPC.O_plasmanyles	Glycerophospholipids	1-Monoetherglycerophosphocholine O_plasmanyles	0.886	-0.174	**4.81E-02**	0.902	-0.149	**3.47E-02**

Data are represented by color codes for log_2(fold-change) and paired Student's t-test p-values. FC: fold change

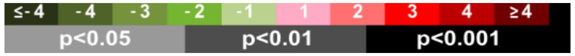

Table S3. Heatmap representing individual metabolic compounds significantly altered at 12 weeks or 24 weeks of receiving RPV/FTC/TDF compared to baseline values.

Metabolite	Metabolite Class	Individual notation	12 weeks *versus* baseline			24 weeks *versus* baseline		
			**FC**	**log$_2$(FC)**	**P-value**	**FC**	**log$_2$(FC)**	**P-value**
Lipids	All the lipids	PC+PE+LPC+LPE+PI+LPI+PS+PG+LPG+DAG+TAG+NEFA+SL	0.894	-0.162	**5.14E-02**	0.888	-0.172	**4.28E-02**
Total NEFA	Non-esterified fatty acids	Non-esterified fatty acids	1.286	0.363	**2.58E-01**	1.475	0.561	**4.83E-02**
NEFA_omega_6	Non-esterified fatty acids	NEFA omega 6	1.520	0.604	**7.84E-02**	1.660	0.731	**2.18E-02**
SFA	Non-esterified fatty acids	Saturated fatty acids	1.229	0.297	**5.79E-01**	1.376	0.460	**3.83E-02**
FFA.16.1	Non-esterified fatty acids		2.047	1.034	**1.33E-01**	2.818	1.495	**2.94E-02**
FFA.18.2	Non-esterified fatty acids		1.577	0.657	**6.13E-02**	1.713	0.777	**3.55E-03**
HODE	Oxidized fatty acids	Hydroxy-octadecadenoic acids	1.419	0.505	**4.23E-02**	1.238	0.309	**4.80E-02**
AC	Acylcarnitines	Acylcarnitines	1.834	0.875	**3.04E-03**	1.882	0.913	**8.41E-04**
DAG	Glycerolipids	Saturated Diacylglycerols	0.868	-0.205	**2.50E-02**	0.879	-0.186	**8.58E-02**
TAG	Glycerolipids	Triacylglycerols	0.803	-0.316	**1.92E-02**	0.744	-0.427	**5.25E-03**
Lipids Storage	TAG+ChoE	TAG+ChoE	0.797	-0.328	**1.22E-02**	0.740	-0.435	**4.14E-03**
ChoE	Sterols	Cholesteryl Esters	0.852	-0.231	**3.76E-02**	0.793	-0.335	**1.78E-02**
ST	Sterols	Steroids	1.450	0.536	**1.61E-01**	1.565	0.646	**2.60E-02**
DAPE	Glycerophospholipids	Diacylglycerophosphoethanolamine	0.852	-0.230	**2.84E-02**	0.904	-0.146	**7.05E-02**
DAPC	Glycerophospholipids	Diacylglycerophosphocholine	0.788	-0.343	**6.67E-03**	0.778	-0.362	**1.11E-02**
PC	Glycerophospholipids	Phosphatidylcholines	0.846	-0.241	**1.88E-02**	0.820	-0.287	**1.46E-02**
PC.DHA	Glycerophospholipids	PC-DHA	0.813	-0.298	**3.05E-02**	0.826	-0.275	**4.76E-02**
PC_20.4	Glycerophospholipids	PC 20:4	0.885	-0.176	**5.64E-02**	0.861	-0.217	**3.43E-02**
PC_PEMT	Glycerophospholipids	PE PEMT	0.865	-0.209	**4.00E-02**	0.840	-0.252	**3.60E-02**
Chol_PC	Cho01 and PC		0.846	-0.241	**1.88E-02**	0.820	-0.287	**1.46E-02**
Cer	Sphingolipids	Ceramides	0.928	-0.108	**1.72E-01**	0.875	-0.193	**2.03E-02**
Cer+PC	Ceramides and PC		0.850	-0.235	**2.01E-02**	0.822	-0.283	**1.41E-02**
SM	Sphingolipids	Sphingomyelins	0.943	-0.084	**1.87E-01**	0.893	-0.163	**3.07E-02**
SL	Sphingolipids	Cer+SM+CHM+FSB	0.954	-0.069	**2.76E-01**	0.896	-0.159	**2.53E-02**
SM+DAG	SM and DAG		0.914	-0.130	**1.02E-01**	0.861	-0.216	**1.42E-02**

Data are represented by color codes for log$_2$(fold-change) and paired Student's t-test p-values. FC: fold change

≤-4	-4	-3	-2	-1	1	2	3	4	≥4

p<0.05	p<0.01	p<0.001

Table S4. Heatmap representing significantly altered metabolites obtained for the comparison between 24 weeks to 12 weeks in patients of each group.

Class		Individual annotation	Control			Experimental		
			FC	log_2(FC)	P-value	FC	log_2(FC)	P-value
Non-esterified fatty acids	SFA	18:0	1.214	0.280	2.66E-02			ns
	PUFA	20:3n-9	1.446	0.533	2.30E-02			ns
		-	1.278	0.354	4.58E-02			ns
Fatty esters	AC	AC(12:0)	0.840	-0.251	2.03E-02			ns
		-	0.884	-0.179	3.49E-02			ns
Glycerolipids	TAG	TG(55:4)			ns	0.725	-0.463	4.24E-02
Sterols	ChoE	ChoE(22:6)	0.836	-0.258	1.91E-03			ns
	ST	-			ns	1.806	0.853	4.36E-02
		-			ns	1.783	0.834	4.67E-02
Glycerophospholipids	MEMAPE	PE(P-18:0/18:1)			ns	0.826	-0.276	1.09E-02
		PE(P-18:1/20:4)			ns	0.836	-0.258	3.75E-02
	MAPE	PE(22:6/0:0)			ns	1.157	0.210	4.75E-02
	DAPE	PC(16:0/19:1)			ns	0.876	-0.192	4.89E-03
	DAPC	PC(18:0/18:2)			ns	0.904	-0.145	3.54E-02
		PC(18:2/18:2)			ns	0.839	-0.254	2.87E-02
		PC(36:3)			ns	0.913	-0.132	4.36E-02
		PC(18:2/20:4)			ns	0.922	-0.116	1.94E-02
	MEMAPC	PC(O-16:0/16:0)	0.937	-0.094	4.49E-02			ns
		PC(O-16:0/22:4)	0.930	-0.104	4.91E-02			ns
		PC(O-18:0/20:4)	0.898	-0.155	3.01E-02			ns
		PC(O-22:0/20:4)	0.892	-0.165	2.27E-02			ns
		PC(O-34:1)			ns	0.942	-0.087	2.76E-02
		PC(P-18:0/20:4)	0.888	-0.171	2.01E-02			ns
	MAPC	PC(16:1/0:0)			ns	1.173	0.230	1.33E-02
		PC(20:2/0:0)	1.566	0.647	4.95E-02	1.237	0.307	1.65E-02
		PC(20:4/0:0)			ns	1.150	0.201	2.62E-02
		PC(22:6/0:0)			ns	1.145	0.196	2.25E-02
		PC(0:0/17:1)			ns	1.213	0.278	1.99E-02

		PC(0:0/20:4)			**ns**	1.173	0.230	**2.62E-02**
		PC(0:0/22:4)			**ns**	1.170	0.227	**1.81E-02**
		PC(0:0/22:6)			**ns**	1.125	0.170	**3.54E-02**
	MEPC	PC(O-20:0/0:0)			**ns**	1.102	0.141	**3.67E-02**
		PC(O-24:2/0:0)			**ns**	1.164	0.219	**4.33E-02**
		PC(P-18:1/0:0)			**ns**	1.203	0.267	**3.73E-03**
	MAPI	LPI(16:0)			**ns**	1.256	0.329	**2.58E-02**
		LPI(18:0)			**ns**	1.159	0.213	**1.23E-02**
		LPI(18:0)			**ns**	1.168	0.224	**1.85E-02**
		LPI(20:4)			**ns**	1.144	0.195	**3.13E-02**
**Sphingolipids**	Cer	Cer(d43:1)	0.845	-0.242	**1.28E-02**			**ns**
	SM	SM(33:1)	0.904	-0.146	**2.12E-02**			**ns**
		SM(d18:0/18:0)	0.878	-0.187	**3.96E-02**			**ns**
		SM(36:2)	0.911	-0.134	**3.48E-02**			**ns**
		SM(d18:1/22:0)	0.864	-0.211	**4.46E-02**			**ns**
		SM(d18:1/23:0)	0.897	-0.156	**3.93E-02**			**ns**
		SM(d18:1/24:1) + SM(d18:2/24:0)	0.892	-0.165	**3.58E-02**			**ns**
		SM(d18:2/20:0)			**ns**	0.926	-0.111	**2.45E-02**
		SM(d18:2/22:0)	0.920	-0.121	**4.55E-02**			**ns**
		SM(d18:2/23:0)			**ns**	0.937	-0.094	**3.00E-02**

Data are represented by color codes for log_2(fold-change) and paired Student's t-test p-values. FC: fold change, ns: no statistically significant

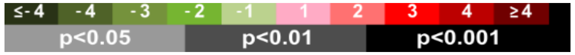

Experimental group was receiving a combination of RPV/FTC/TDF and the control group was receiving a combination of EFV/FTC/TDF during the 24 weeks of the study.

Figure S1. Heatmap representing Pearson's correlations between lipid metabolites obtained by UHPLC-MS and lipid parameters conventionally used in clinical practice at 12 and 24 weeks compared to baseline (B) in patients taking EFV/FTC/TDF (1) or RPV/FTC/TDF (2) in the ART. Blue boxes highlights most relevant significant correlations.

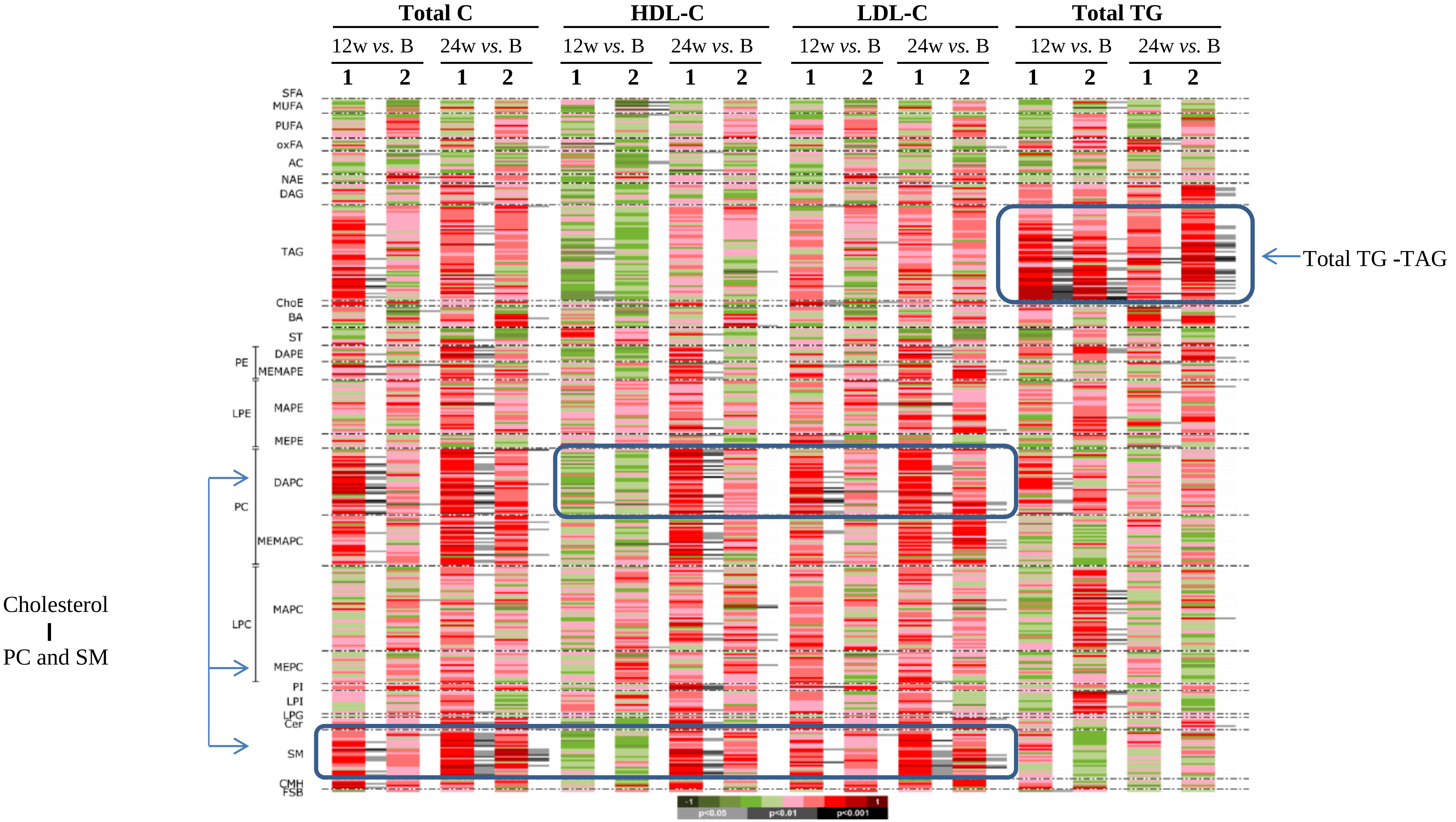

Figure S2. Heatmap representing Pearson's correlations between lipid metabolites obtained by UHPLC-MS and apolipoprotein A (apoA) and apolipoprotein B (apoB) obtained by conventional laboratory measurements at 12 and 24 weeks compared to baseline (B) in patients taking EFV/FTC/TDF (1) or RPV/FTC/TDF (2) in the ART. Blue boxes highlights most relevant significant correlations.

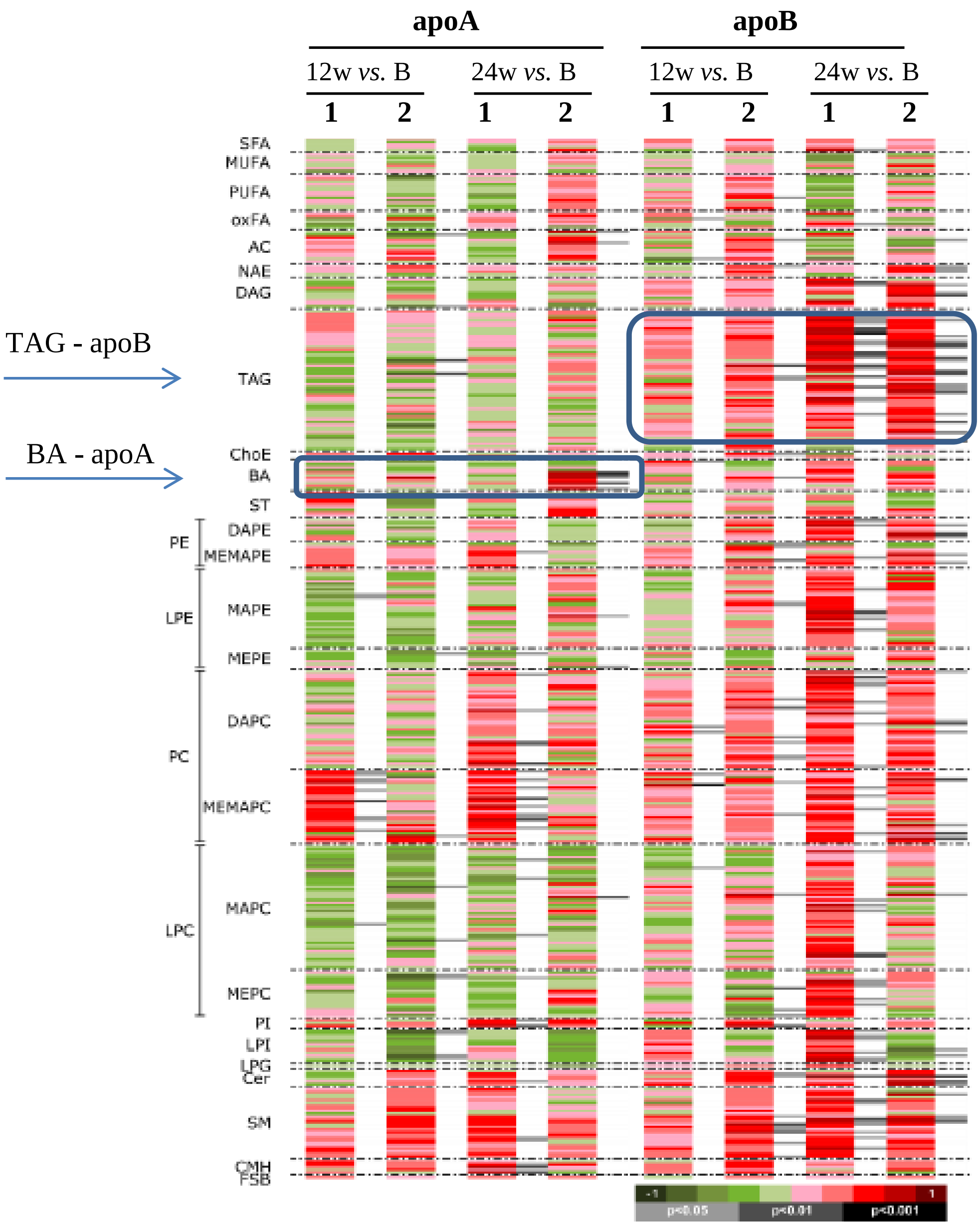

Figure S3. Effect of switching from EFV to RPV in the liver condition. **A**) Evaluation of conventional biochemical markers, **B**) Evaluation of presence and development of non-alcoholic fatty liver disease (NAFLD) based on the OWLiver® Care and OWLiver® test, a non-invasive test using plasma samples, and **C**) Storage lipid changes due to ART randomization

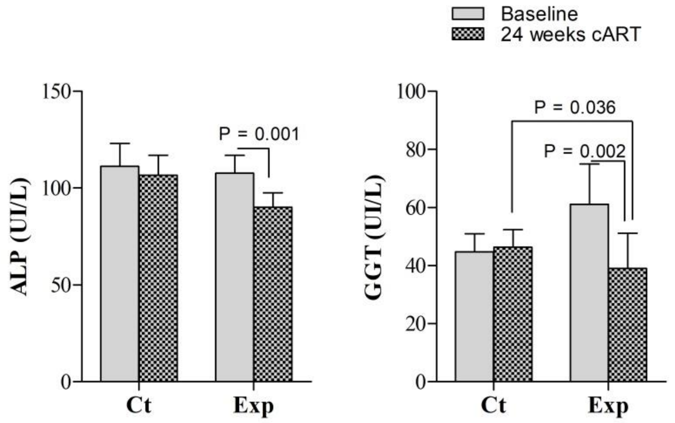

BASELINE	24 weeks of cART	EFV	RPV
**No NAFLD**	No NAFLD	2	2
	Steatosis	2	2
	**NASH**	**2**	**0**
**Steatosis**	No NAFLD	2	0
	Steatosis	4	6
	**NASH**	**0**	**3**
**NASH**	Steatosis	1	0

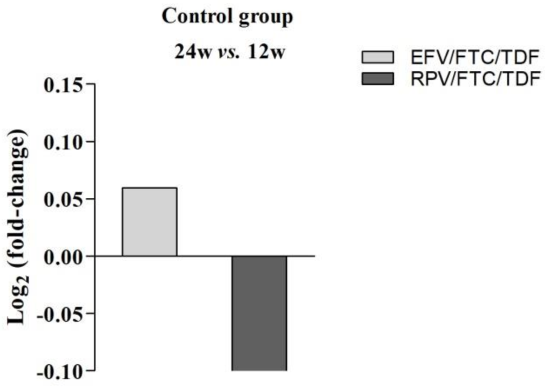

NAFLD, Non-alcoholic fatty liver disease; NASH, non-alcoholic steatohepatitis

Experimental group was receiving a combination of RPV/FTC/TDF and the control group was receiving a combination of EFV/FTC/TDF during the 24 weeks of the study.
